# Effective student engagement with blended learning: A systematic review

**DOI:** 10.1016/j.heliyon.2024.e39439

**Published:** 2024-10-16

**Authors:** M. De Bruijn-Smolders, F.R. Prinsen

**Affiliations:** Research Centre Urban Talent, Rotterdam University of Applied Sciences, Rotterdam, the Netherlands

**Keywords:** Blended learning, Student engagement, Learning outcomes, Systematic review

## Abstract

Although student engagement is known to promote learning outcomes in higher education, what elements of blended learning designs impact effective student engagement and hereby learning outcomes, has not been clarified yet. Hence, it is unknown how to engage students with blended learning in an effective manner. The current study breaks down student engagement into four dimensions (academic, behavioral, cognitive, and affective), and reviews the evidence regarding blended learning that engages students effectively, whether this is academically, personally, socially, or with regard to citizenship. The studies reviewed (*k* = 15, *N* = 1,428) overall asserted that all blended learning interventions investigated had a moderate to high impact on student engagement and on learning outcomes. This review, a summary and insight into the evidence, is important for the field's understanding as well as for professionals in higher education: for lecturers and policy makers who want to introduce and monitor blended learning as a means to promote both student engagement and their learning outcomes in higher education. Further research is required to increase our knowledge of how blended learning impacts both multi-dimensional constructs: student engagement and learning outcomes.

## Highlights

• Blended learning has a moderate to high positive impact on academic, behavioral, cognitive, and affective engagement.
• Blended learning has a moderate to high positive impact on learning outcomes, regardless of its design features.
• Effective designs: a flipped classroom, (an) online: learning community, peer assessment, fun factor, student intervention.
• Blended learning literature generally measures student engagement with respect to academic learning outcomes.
• Blended learning research should study learning outcomes academically, personally, socially, and regarding citizenship.

## 1 Introduction

Student engagement is the energy that students put into their learning [1]. If students are more engaged, they will invest more energy in their learning, resulting in better learning outcomes, which can in succession nourish further engagement [1].

Blended learning, an integration of the strengths of face-to-face teaching (e.g., classroom interaction) and online teaching (e.g., technologically mediated interactions between students, teachers and learning resources) or computer-mediated instruction [[2], [3], [4], [5], [6], [7]], has been found to benefit students’ academic learning outcomes [[8], [9], [10]]. However, whether and how blended learning environments relate positively to student engagement and (therefore) to learning outcomes, not only academically, but also personally, socially, and in relation to citizenship, is yet unclear.

To be able to blend online learning and learning on location in an effective manner, “the best of both worlds”, it is important to know how to blend them, and how such a blend can engage students for successful learning. This knowledge is important for the field's understanding as well as for professionals in higher education. This review aims to provide a summary and insight into the evidence, for lecturers and policy makers who want to introduce and monitor blended learning as a manner to promote student engagement and hereby learning outcomes in higher education.

To summarize, student engagement has shown to benefit students' academic learning outcomes; however, it is yet unknown whether and how *blended learning* in higher education engages students effectively; academically, personally, socially, and with respect to citizenship. Consequently, the central research question of the current review is: To what extent and how is blended learning beneficial both for student engagement and (consequently) for students’ learning outcomes, academically (e.g., grades, grade-point average), personally (e.g., well-being), socially (e.g., working with others), and with respect to citizenship (e.g., participatory citizenship)?

With this goal, the empirical literature with regard to blended learning, student engagement, and learning outcomes will be systematically reviewed. Previous to specifying this systematic review's method, it is first necessary to briefly describe effective blended learning and student engagement.

### 1.1 Effective blended learning

A meta-analysis on 50 effects found in 40 studies published between 1997 and 2008 concluded that students in blended learning, a combination of online learning and face-to-face learning, modestly outperformed (academically) their fellow students, who engaged in face-to-face instruction [9]. The authors also found in their 2013 meta-analysis that pedagogical approach moderated the impact of online learning; collaborative and expository instruction both had a significant effect, while independent learning did not.

In 2015, a meta-analysis on 56 blended learning studies and academic outcomes focusing on health professions also confirmed that blended learning consistently and positively impacted knowledge acquisition, and had more or at least the same effect as non-blended instruction (versus online learning or face-to-face learning) [8]. With respect to blended learning, Liu et al. [8] found that both the presence of exercises and the usage of objective assessments (versus subjective assessments) yielded larger effect sizes for blended learning.

Finally, in 2017 a meta-analysis of blended learning at the course level in higher education showed that blended learning is a more beneficial alternative to face-to-face learning with respect to final course grades [10]. However, it was also found that students with high grade-point averages benefit especially from blended learning, as do students who study science, technology, engineering, and/or mathematics (STEM) [10]. The authors assumed that the concrete linear learning of STEM-students who learn to apply and test ideas, based on grounded theory, guided by more direct teaching, might be supported more easily with blended learning compared to the learning of non-STEM students, who learn to take into account more perspectives, requiring more online constructive dialogues, peer-to-peer interaction, and more indirect support by their tutors [10].

Bond et al. [1] systematically mapped research from 243 studies of student engagement and educational technology in higher education published between 2007 and 2016. Of the 204 studies in which the mode of delivery had been indicated, 109 studies used blended learning. Bond et al. [1] concluded that research had mainly been done on the behavioral aspect of engagement, not including cognitive and affective engagement. The authors limited their review to types of educational technology and indicators of student engagement that had been researched, including achievement as one of the indicators for engagement; they did not explore to what extent blended learning might impact either student engagement and/or (subsequently) learning outcomes.

In summary, although several meta-analyses have concluded that blended learning has a positive impact on students’ academic learning outcomes [[8], [9], [10]], it is yet difficult to determine what exactly constitutes effective blended learning designs in higher education. To what extent and how students can be effectively engaged, for promoting learning outcomes (academically, personally, socially, and with respect to citizenship), and by which blended learning designs is not yet known.

### 1.2 Present study

In the current study, we investigate whether and how blended learning has an effect both on student engagement, and (consequently) on their learning outcomes. Meta-analyses until now have shown that different design features of blended learning have an effect on students’ academic learning outcomes [[8], [9], [10]]. However, it is as yet difficult to identify which specific design features of blended learning matter most [6]. In their review of research on blended learning studying outcomes with regard to aspects such as motivation, efficiency, and costs, Terbeek et al. [6] were able to compare the studies (*k* = 10) on one, two or three of the following criteria: technology, teaching method, and learning activity. The authors concluded that future blended learning studies might become more comparable with each other if more attention is paid in blended learning research on the educational design of blended learning, as well as on the traditional (face-to-face) learning context with which blended learning has been compared. For the purpose of gaining more insight into the elements of blended learning design that might impact student engagement and learning outcomes, the current review will describe the different designs in detail, for the experiment group, the control group, and the blended learning design will be categorized by the authors.

To provide an idea of learning designs that might have an effect on student engagement and on learning outcomes, we briefly describe three well-known blended learning designs that are claimed to have such effects: online learning communities, flipped classrooms, and gamified learning.

An *online learning community* is a supportive virtual environment in which a group of learners unite by a common cause to engage in collaborative learning [11]. Learning communities that enable students connecting with their fellow students, sharing knowledge with one another, and achieving common goals, can reduce dropout rates [12]. The instructor–learner interaction and peer interaction in learning communities might benefit course performance as well as course satisfaction [13]. Social presence (the feeling of “being with another”) [14] and teaching presence are crucial elements for an online learning community to have an effect on students’ learning [15].

In a *flipped classroom*, students study lesson material prior to class for applying this during class. Flipped classrooms are concluded to generate significantly better learning outcomes contrasted to face-to-face instruction. However, critical features for outcomeful implementation are maintaining face-to-face learning time and using quizzes [16]. In line with Van Alten et al. [16], we believe that instructional material studied before class can be in any form, including physical materials, books, or hard copy hand-outs. However, in the current review, studies on flipped classrooms are only included if the instructional materials studied before class are online, followed by face-to-face learning in class.

*Gamified learning frameworks*, an integration of game-based design elements, and numerous gamification forms in higher education have been found to benefit students in a number of ways, think of improving student engagement, motivation, confidence, attitude, perceived learning, and performance [17]. In their systematic review based on 18 studies, the authors concluded that increased student attitudes, student engagement and performance were most significantly benefited by gamified learning.

### 1.3 Student engagement in learning

Active research on student engagement bloomed in the 1980's. Since then, an extensive knowledge base on indicators of student engagement for improved academic achievement has been built. At first, the literature focused mainly on the length of time for which students would remain academically engaged. Later on, additional dimensions of student engagement became important, such as students' behavior, emotion, and cognition [18]. Student engagement research lacks consensus on the number of subtypes of student engagement and its definitions: there can be identified student engagement models with two-, three-, and four-subtypes [19]. To be able to compare research addressing such broad concepts as effective blended learning and learning outcomes, the meta-construct of student engagement, consisting of the four dimensions identified by Appleton, Christenson, Kim, and Reschly [20], and Christenson et al. [21] has been chosen to guide this review. These authors distinguished the following four dimensions of student engagement: 1) academic engagement (e.g., time on task, homework completion), 2) behavioral engagement (e.g., attendance, participation), 3) cognitive engagement (e.g., self-regulation, value/relevance) and 4) affective engagement (e.g., sense of belonging, motivation).

Below, the research questions of this review are outlined.

## 2 Research questions

To what extent can blended learning stimulate student engagement that promotes learning outcomes?

1. To what extent does blended learning influence student engagement in learning, along with learning outcomes, and what are hindering factors?
2. To what extent does student engagement relate to learning outcomes, in the context of blended learning?

**Fig. 1:**
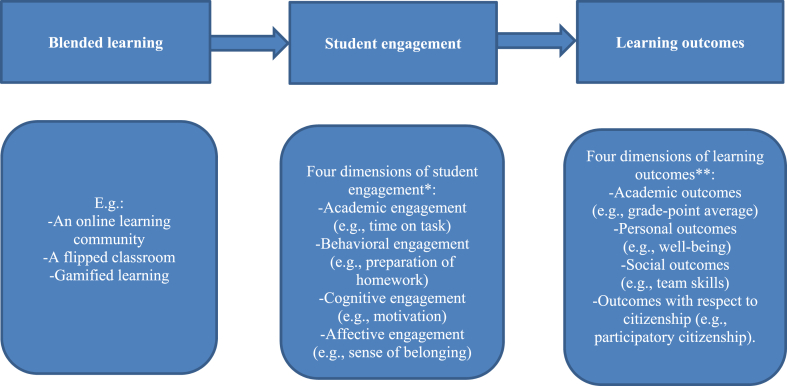
Conceptual model blended learning review *∗* In line with Appleton et al. [20], and Christensen et al. [21] ∗∗ In line with Conway et al. [23].

In Fig. 1 this review's two research questions are visualized.

## 3 Method

### 3.1 Procedure

In the current study, we used Petticrew and Roberts's [22] method for performing systematic reviews. The process of conducting this review had four phases. First, the inclusion criteria of studies were formulated. Next, the relevant search terms and databases were determined. Then, the literature was searched extensively and studies were selected for inclusion. Finally, effect sizes were computed, generating standardized data by which the included studies' results could be compared. The results are described and summarized according to their blended learning design, which is linked with learning outcomes.

The studies reviewed were found to be highly heterogeneous with respect to student engagement. For this reason, it was not able to conduct a meta-analysis. Instead, the different effect sizes were computed, not the mean effect. On top of the variables that were measured quantitatively by the authors in the included studies, the studies indicated that blended learning had had other impacts on learning outcomes, based on the authors' conclusions and on students' self-reports. To learn more about the effect of the studies on student engagement and learning outcomes, the authors' conclusions and the students’ self-reports are also described in our results.

### 3.2 Inclusion and exclusion criteria

1. *Research scope:* This review concentrates on the impact of learning interventions in a blended learning environment in higher education on student engagement and on learning outcomes. We felt it important that the engagement and learning outcomes occurred in an existing curriculum researched with field studies, not with laboratory studies.
2. *Blended-learning interventions:* The blended-learning interventions under research in this systematic review aimed at enhancing both student engagement and their learning outcomes in blended learning, in the context of higher education. As a consequence, studies that solely researched either student engagement or learning outcomes were not included.
3. *Learning outcomes:* In line with Conway et al. [23], we distinguish four dimensions of learning outcomes: academic (e.g., knowledge, grade-point average), personal (e.g., well-being, moral development), social (e.g., team skills, understanding and tolerating diversity), and with respect to citizenship (e.g., participatory citizenship).
4. *Student characteristics:* One of the aims of the current review is generalizing the results to higher education learning. Therefore, included studies should involve (post-) tertiary education students who are representative of the general school community. Subsequently, students with special characteristics (e.g., labeled as excellent or gifted) were not included in this review.
5. *Research design:* To assure a methodological standard, the design in the studies included had to be either 1) an experimental pretest-posttest design including a control group, whereby students had to be randomly divided between the experimental and control group, or 2) a quasi-experimental pretest-posttest design with a control group, in which students were not randomly selected for either the experimental or control group. Instead, existing groups of students (e.g., classes) were compared or students were divided between the experimental and control group based on certain conditions (e.g., a preference for the intervention in particular). Studies that lacked a (quasi-) experimental design, including a control group, were excluded.
6. *Results:* For the comparison and generalization of results, the data had to be quantitative, including effect sizes or presenting information that allowed effect sizes to be computed.
7. *Quality of the study:* Publication of the studies had to be in English, and had to been peer-reviewed and listed in the Social Science Citations Index (Expanded). Finally, the study had to report results accurately, for example, stating the number of participants in both the experimental and control group(s).

### 3.3 Databases and search terms

The most well-known databases for educational research were explored via EBSCOhost, a search engine by which a great many databases can be sampled at the same time for example, Pubmed, Cinahl, Eric, Psychology and Behavioral Science Collection, and Academic Search Ultimate. The search string used consisted of the search terms: blended learning OR hybrid learning AND higher education AND experiment∗. To narrow the search as much as possible, the boxes “multi-disciplinary”, “medical”, and “education”, were ticked. In addition, for blended learning or hybrid learning “abstract” was selected, for higher education and experiment∗ AT (all text) was selected.

### 3.4 Selection of studies and data extraction

**Fig. 2:**
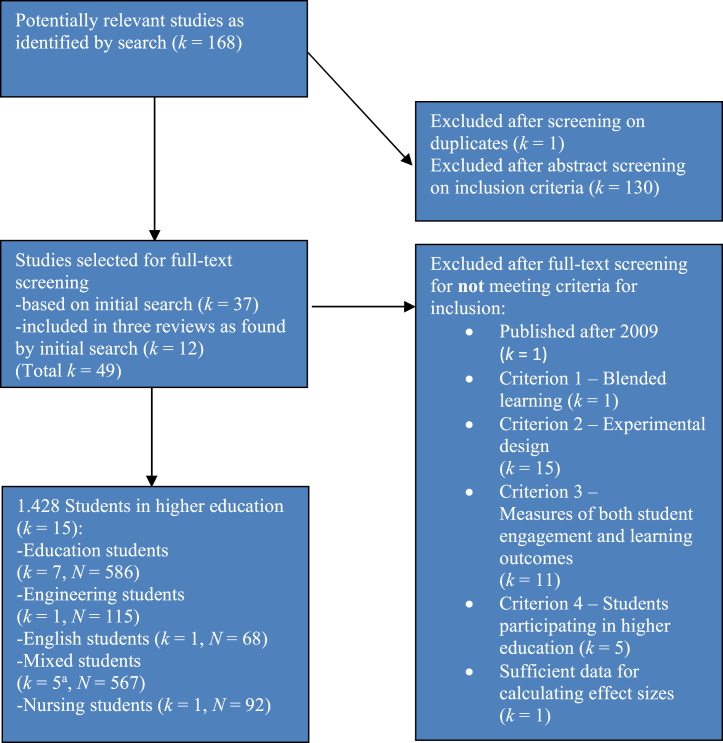
Study selection process ^a^ One of these studies consisted of two experiments

The study selection and the data interpretation were done independently by both the first author and a research assistant, using a self-developed data-extraction form. If an included study concerned a meta-analysis, the studies included in that particular meta-analysis were selected and interpreted also. Thus, in the current study, studies were selected directly by a search string, and indirectly via primary studies selected (snowball method). The first author trained the research assistant in using the self-developed data-extraction form. An inter-rater reliability of 90 % was achieved for the final study selection. The remaining 10 % of the selected studies were carefully discussed due to divergent individually obtained results, resulting in consensus. See Fig. 2 for the study selection process.

### 3.5 Coding of outcome measures

The different student engagement measures researched in the included studies were compared, categorized and labeled using four dimensions of student engagement: 1) academic engagement (e.g., time on task, homework completion), 2) behavioral engagement (e.g., attendance, participation), 3) cognitive engagement (e.g., self-regulation, value/relevance) and 4) affective engagement (e.g., belonging, identification with school) [21,22]. For learning outcome measures we chose to formulate labels (e.g., student well-being, final examination score) ourselves after comparison and categorization based on the following four dimensions of learning outcomes: academic (e.g., grade-point average, cognitive outcomes), personal (e.g. well-being, moral development), social (e.g., interacting with others, tolerating diversity), and with respect to citizenship (e.g., participatory citizenship) [54].

### 3.6 Effect size computations for student engagement and learning outcomes

The coded outcome variables were quantified in a standardized manner, by the usage of effect sizes, for two reasons. The first was to ensure that the different outcome variables regarding student engagement and learning outcomes could be contrasted. The second was to evaluate the impact of a blended learning intervention. In specific for studies with a small number of participants, an effect size may indicate that, although criterial significance was not achieved, there could still exist an impact of the learning intervention. The effect sizes were either extracted of the included studies, or, if not present, were computed (see also [24,25]). If an effect size was not present, the effect size was computed by calculating the mean difference between the treatment group and the control group, divided by the pooled standard deviation. This so called standardized mean difference is Cohen's *d* [26]. If the means and standard deviations were not reported in the studies included, effect sizes were computed using the formulas from Lipsey and Wilson [27]. In line with Cohen [26], effect sizes were concluded to be low (.20 < *d* ≤ .50), moderate (.50 < *d* ≤ .80), or high (*d* ≥ .80).

## 4 Results

### 4.1 Overview of the included studies

12 out of the 15 included studies compared a blended learning environment (treatment group) with a face-to-face learning environment (control group). The three remaining studies conducted research on a specific design feature of blended learning, specifically, game mechanics [30] with two experiments [[30a], [30b]], augmented reality [40], and an individualized intervention approach [41].

From a technical point of view, the blended learning environments in each study differ to a striking extent. For example, studies used different media for creating online learning communities (e.g., Edmodo, Google drive, the YouTube platform). However, from a didactic point of view, the blended learning designs in the different studies can be categorized according to the main aspects of learning they were meant to boost. The included studies are described below, grouped by whether their blended learning design involved.

a) an online learning community of students and tutors to promote peer learning and student–tutor interaction [28,29,31,32,34,35,39,42];
b) a flipped classroom in which students prepare their lessons online, (e.g., by videos or podcasts), and then participate in offline and online application tasks in the classroom, mostly in groups of students, to promote self-regulated learning [33,36,37];
c) online peer assessment as a necessary part of students' learning process [38];
d) an online fun factor meant as an incentive for students to learn more and to work harder (e.g., handing out badges or medals online, or by using Augmented Reality) [30a,30b,^40^];
e) an individualized intervention approach in which students were provided with a monthly report of their online and offline learning behavior, along with a customized intervention [41].

The effects of each blended learning design (online learning community, flipped classroom, peer assessment, online fun factor, and individualized intervention approach), are further described below, as measured with regard to engagement (academic, behavioral, cognitive, and affective) and learning outcomes (academic, personal, social, and with respect to citizenship). On top of the *measured* effects, *experienced* effects reported by the authors or the students involved will also be described below. Additionally, factors hindering outcomeful implementation that were mentioned by the authors will be described. For an overview of measured effects and a detailed overview of each study included, please see Table 1, Table 2, respectively.

**Table 1:** Aspects of student engagement and learning outcomes examined in included studies

	Engagement											Learning outcomes				
	Academic	Behavioral		Cognitive							Affective	Academic			Personal	Social
Included studies (in alphabetical order)	Quality of assignment	Interactive learning	Number of absence days	Critical thin-king	Learning Satis-faction	Learning perceptions	Motivation	Attitudes	Sense of efficacy	Self-confidence in learning	Sense of community	Academic achievement	Final exam score	Recall factual knowledge	Well-being	Presenting work
[28]					✓						✓		✓			
[29]	✓											✓				
[30a]		✓												✓		
[30b]		✓												✓		
[31]								✓							✓	✓
[32]							✓					✓				
[33]									✓				✓			
[34]								✓				✓				
[35]		✓										✓				
[36]						✓						✓	✓			
[37]	✓		✓										✓			
[38] a				✓								✓	✓			
[39]						✓						✓				
[40]					✓							✓				
[41]							✓	✓						✓		
[42]					✓					✓		✓				

a Only Experiment 1 of the 2 experiments in this article was included in the current review. Experiment 1 only used student engagement measures, not measures regarding learning outcomes.

**Table 2:** Overview of the included studies (in alphabetical order).

No	Participants	Research design, Treatment group	Control group	Student Engagement	d	Effect d	Measurement of engagement	Measurement of learning outcomes	d	Effect d
[28]	Education students (*N* = 140)	Quasi-experimental design Online learning community (*n* = 81)	Traditional face-to-face learning (*n* = 59)	*Cognitive engagement* - learning satisfaction *Affective engagement* - sense of community	.41 .48	+ +	Students' rating of instruction [43] Sense of Classroom Community Scale [44]	*Academic learning outcome* Final examination score	1.35	+++
[29]	Education students (*N* = 44)	Experimental design Online learning community (*n* = 22)	Face-to-face learning (*n* = 22)	*Academic engagement* - multimedia design scores - visual design scores	1.19 .65	+++ ++		*Academic learning outcome* Achievement test for course	.47	++
[30a]	Mixed students (*N* = 22)	Experimental design Experiment 1 Online fun factor: gamified blended learning (*n* = 11)	Non-gamified blended learning (*n* = 11)	*Behavioral engagement* Forum messages posted	3.19	+++	Meant to boost self-determination Application tasks (need for competence) [45]	*Academic learning outcome* Recall of factual knowledge	.62	++
[30b]	Mixed students (*N* = 42)	Experiment 2 Online fun factor: gamified blended learning (*n* = 20)	Non-gamified blended learning (*n* = 22)	*Behavioral engagement* Forum messages posted	1.42	+++		*Academic learning outcome* Recall of factual knowledge	−.45	–
[31]	Engineering students (*N* = 115)	Quasi-experimental design Online learning community (*n* = 57)	Face-to-face learning (*n* = 58)	*Cognitive engagement* Overall attitudes toward the cooperative learning activities	.52	++		*Personal learning outcomes* Feeling peer pressure … -when presenting my work -when commenting on others' work Feeling time pressure *Social learning outcome* Presenting work	1.04 .48 .92 .56	+++ + +++ ++
[32]	Education students (*N* = 60)	Experimental design Online learning community: (*n* = 30)	Face-to-face learning (*n* = 30)	*Cognitive engagement* Learning motivation	.93	+++	Motivated Strategies for Learning Questionnaire [47]	*Academic learning outcomes* Computer technology skills test -Overall -Analysis -Inference -Evaluation -Induction -Deduction Computer technology disposition test -Overall	.80 1.34 .68 .56 .69 .65 .57	++ +++ ++ ++ ++ ++ ++
[33]	Education students (*N* = 62)	Quasi-experimental design Flipped classroom (*n* = 32)	Face-to-face learning (*n* = 30)	*Cognitive engagement* Sense of self-efficacy in … Student engagement Instructional strategies Classroom management	.35 .29 .26	+ + +	Teacher Sense of Efficacy Scale [46]	*Academic learning outcome* Final exam scores	.94	+++
[34]	Mixed students (*N* = 30)	Experimental design Online learning community (*n* = 15)	Face-to-face learning (*n* = 15)	*Cognitive engagement* Attitudes -Total score	2.87	+++	Students' learning attitudes questionnaire (self-developed by the authors)	*Academic learning outcomes* Exam scores Students' professional skills test (self-assessment) -Total score	.77 2.18	++ +++
[35]	Mixed students (*N* = 66)	Quasi-experimental design Online learning community (*n* = 24)	Control group 1 Face-to-face learning (*n* = 26) Control group 2 Face-to-face learning (*n* = 16)	*Behavioral engagement* Interactive learning Time spent on … -Facebook -Skype -Wiki -Discussion Forums	.58 .48 .45 .52	++ + + ++		*Academic learning outcome* University-wide English placement test, designed by English department	**-∗**	**-**
[36]	Mixed students (*N* = 43)	Experimental design Flipped classroom (*n* = 24)	Face-to-face learning (*n* = 19)	*Cognitive engagement* Student perception of the course structure -Aims -Demanding -Well organized -Clear -Effective respons -Instructor enthusiasm -Course sign -Feedback -Quality -Instructor effectiveness	.71 −.03 .31 .79 1.21 .63 .75 1.77 1.16 .94	++ – + ++ +++ ++ ++ +++ +++ +++	Likert-items, self-developed by the authors	*Academic learning outcome* Grade-point average Cumulative final exam score	−.45 .75	– ++
[37]	Education students (*N* = 128)	Quasi-experimental design Flipped classroom (*n* = 64)	Face-to-face learning (*n* = 64)	*Academic engagement* **-**Project quality *Behavioral engagement* -Number of online training courses attended -Number of ICDL tests taken -Number of days absent	.95 1.04 .89 1.30	+++ +++ +++ +++		*Academic learning outcome* Final grade	.88	+++
[38]	Experiment 1∗∗ Mixed students (*N* = 364)	Experimental design Online peer assessment (*n* = not reported)	Face-to-face learning (*n* = not reported)	*Cognitive engagement* Applied critical thinking	.13	0	Applied Critical Thinking measure [48] Questions on course content	*Academic learning outcomes* Final exam Critical thinking exam question Scores of the final exam Questions on course content	.50 .24 .13	+ + 0
[39]	English students (*N* = 68)	Quasi-experimental design Online learning community (*n* = 38)	Face-to-face learning (*n* = 30)	*Cognitive engagement* Learning perceptions -Personal relevance or importance of the course	.22	+	Personal involvement inventory (PII) [49], adapted by the author	*Academic learning outcome* Microsoft Excel certificate examination	.53	++
[40]	Education students (*N* = 103)	Quasi-experimental design Online fun factor: Augmented reality (AR)-based blended strategy (*n* = 59)	Face-to-face learning (*n* = 54)	*Cognitive engagement* Learner feedback on using the different learning supports Q1 Q2 Q3 Q4 Q5 Q6	.72 −.99 −.19 −.73 −.13 .58	++ – – – – ++		*Academic learning outcomes* Grades for weekly learning -Week 1 -Week 2 -Week 3	−.27 .28 −.59	– + –
[41]	Education students (*N* = 49)	Experimental design Individualized intervention approach in blended learning (*n* = 27)	Undifferentiated intervention approach in blended learning (*n* = 22)	*Cognitive engagement* Learning motivation Attitude	1.33 .64 1.59	+++ ++ +++	Questionnaire on learning motivation, learning, attitude -self-efficacy [[51], [52], [53]], modified by the authors. Students' online learning data; online time, resource utilization, and forum access; students' in-class behavioral data	*Academic learning outcome* Knowledge test for the course [53,54]	.61	++
[42]	Nursing students (*N* = 92)	Quasi-experimental design Online learning community (*n* = 46)	Face-to-face learning (*n* = 46)	*Cognitive engagement* Learning satisfaction Self-confidence in learning	.39 .40	+ +	Student Satisfaction and Self-Confidence in Learning Scale [50]	*Academic learning outcome* Assessment of student-generated video	.60	++

∗Study 30 consists of two experiments. Both experiments are included as two different studies in this review: [30a] and [30b].

∗∗Experiment 2 not included: Only student engagement was researched, not learning outcomes.

Note. Effect sizes were considered negative (d ≤ .00), zero (.00 < d ≤ .20), small (.20 < d ≤ .50), moderate (.50 < d ≤ .80), or high (d ≥ .80). Effect sizes are in the Table are labeled as - (negative), 0 (zero), + (small), ++ (moderate), or +++ (high).

### 4.2 Online learning community

In 8 of the 15 included studies, online learning communities were established between students and their tutors in order to support them with an online structure, enabling students to study and learn in cooperation with their fellow students and in interaction with their tutors [28,29,31,32,34,35,39,42]. In these online learning communities, supported through 10.13039/100006785Google Services or Moodle and 10.13039/100005801Facebook, for example, all learning resources were shared by students and their tutors (e.g., videos, animations, and assignments), and an online announcement page was used to facilitate learning activities (e.g., previewing a chapter, participating in peer discussions, completing exercises, interacting with tutors). In studies [39,42], students were allocated to teams of 5–6 students for interactive learning with their peers. No other included studies mentioned whether students were divided into subgroups.

#### 4.2.1 Measured and reported positive effects on student engagement and learning outcomes

Chen and Chiou [28] measured cognitive and affective engagement in hybrid learning among 140 sophomores who engaged in a course called “Evaluation of child development”. Students in the experimental group (*n* = 81) used an e-learning system that provided the following services: posting of materials, submitting assignments, filling in questionnaires, and participating in discussions (teacher–student, or peer-to-peer). Students in the control group engaged in traditional, face-to-face learning. Although in this study blended learning had only small positive effects on students' learning satisfaction (cognitive engagement) and their sense of classroom community (affective engagement), it had a high positive impact on students’ final examination score (academic outcome).

In addition to the effects they had measured, the authors concluded that the blended learning environment had been convenient for students to use (cognitive engagement) and had a positive effect on their general learning motivation (cognitive engagement), presumably through the peer learning that was initiated [28]. The authors also assumed that reflective thinking (academic outcome) had been promoted by the peer feedback the students had given.

Demirer and Sahin's study [29], which used a combination of online (50 %) and classroom sessions (50 %), showed a moderate to high positive influence of blended learning on the quality of assignments (academic engagement) of 22 undergraduate students who participated in an educational technology course, and a moderate positive influence on their scores on the test for the course (academic outcome). The 22 students in the control group solely engaged in classroom sessions.

In Huang et al.’s study [31], both the 57 students in the treatment group and the 58 students in the control group participated in a jigsaw learning activity (named after the idea of a jigsaw puzzle; each student contributes a piece and together they make up the whole). All 115 students (treatment and control group) received lessons and topic assignments in class, followed up by individual study outside of class. After that, students shared their notes and knowledge in an expert meeting with the class. Students in the treatment group used a weblogging system to present their work, give peer feedback and receive feedback from a teaching assistant and from a tutor in a ‘jigsaw meeting’. In the control group, students conducted this jigsaw meeting in a classroom; they received feedback on paper from their teaching assistant and instructor. In this study [31], the blog-assisted jigsaw method had a moderate positive impact on students' attitudes (cognitive engagement) and academic achievement, measured by their grades for their presentations (social outcome), compared to students participating in traditional learning. The weblog-assisted jigsaw method also had a high positive impact on students' feeling of peer pressure when presenting their work to their fellow students and on their feeling of time pressure, and a small positive impact on students' feeling of peer pressure when commenting on their peers' work (personal outcome), compared to the students who worked with the traditional jigsaw method.

Jou et al. [32] found a high positive influence on motivation (cognitive engagement) when Google Drive was used for online discussion exercises (versus offline discussion exercises) and a moderate impact on academic achievement (academic outcome) among a sample of 60 undergraduate students participating in a mechanical design course. In this study [32], the authors used a knowledge transformation model to design their blended learning environment.

1. Socialization by means of an interactive online community course.
2. Externalization of tacit knowledge as explicit knowledge by using media.
3. Combination of students and their tutors for presenting and sharing of knowledge via weblogging.
4. Internalization of the “rules” needed for outcomeful cooperation with peers, by using Google sites meant to support students in doing so.

Min et al. [34] found that an online learning community encouraged a sample of undergraduate students (*n* = 30) to work hard and to overcome learning difficulties, compared to students participating in traditional learning. This online learning community had a moderate-high positive impact on students’ attitudes (cognitive engagement) and their achievement (academic outcome).

Another study by Monteiro and Morrison [35] showed a small to moderate positive impact on interactive learning (e.g., time spent on discussion forums and behavioral engagement), and a small positive impact on students’ perceived personal relevance for the course on Applied Information Technology (cognitive engagement). Students reported that an online learning community (labeled by the authors as “collaborative blended learning”) helped them to open their minds, observe situations from different perspectives, and better their time-management, team-work, self-reflection, and reasoning skills. It must be noted that although this study [35] showed a high positive impact of blended learning on both academic and behavioral engagement, it unexpectedly showed no impact of blended learning on a university-wide English placement test. The authors assumed that the duration of this particular experiment had been too short to have an effect: this study concerned a short-term intervention of 9 weeks.

In another study by Tsai [39], the blended learning design concerned an online learning community, “web-based co-regulated learning” (CRL). The authors concluded that CRL had a small impact on students’ learning perceptions (cognitive engagement), and a moderate positive impact on their Microsoft Excel certificate examination (academic outcome), among a sample of 68 undergraduate students who took a course on Applied Information Technology. On top of the measured effects, in this study [39], students reported that 10.13039/100013232CRL had a positive impact on their problem-solving and co-working skills (social outcome), boosted by the regular peer-to-peer discussions and the possibility of contacting teammates for support. The students in this study also claimed that CRL had stimulated them to work harder and to hand in assignments before the deadline (academic engagement) and had developed their skills at “learning by doing” (academic outcome). Finally, these students reported that CRL had taught them to depend less on their teacher (academic outcome).

In a study by Zhang et al. [42], Advanced Nursing Practitioner (ANP) tutors recorded their daily work in Blackboard. Subsequently, both the chair and assistant professor provided feedback on the recorded videos, and moderated peer discussion and peer learning activities in an online learning community of 92 ANP master's students. Students in both the experimental group (engaging in the online learning community, *n* = 46) and the control group (face-to-face tutoring, *n* = 46) generated a 7-min video wherein they showed to have learnt the ANP concepts as taught in both groups. Participation in the online learning community had a small positive impact on both learning satisfaction and on self-confidence in learning (cognitive engagement), and a moderate positive impact on the assessment of students' self-generated videos (academic performance). Finally, in the study [42], in which two aspects of cognitive engagement had been measured (learning motivation and attitude), a third effect on cognitive engagement had been reported by the authors (self-efficacy).

#### 4.2.2 Reported hindering factors

In Jou et al.’s study [32], the authors reported that not all students possessed a tablet, personal computer, or smart phone. Although most students nowadays are skilled in working with ICT-applications, the authors stipulated that it is necessary to train students on using web applications for educational purposes. In Min et al.’s study [34], it was mentioned that sufficient support from the tutors involved was essential, in particular with respect to students who underperform. In Monteiro and Morrison's study [35], it was reported that students hesitated for a long time before starting to learn in the new collaborative manner that was proposed. Both studies [32,35] concluded that students must learn how to engage in blended learning. It was reported in two of the studies [34,35] that sufficient support of tutors in blended learning environments is vital. According to Monteiro and Morrison's study [35] tutors should not assume that students are fully equipped for working together effectively in an online learning community. These authors also described the importance of tutors training their students in the co-working skills needed for an online learning community, for example, leadership, teambuilding, debating, team support, providing feedback and articulating demands.

### 4.3 A flipped classroom

Studies by Kurt [33], Peterson [36], and Safar and Alkhezzi [37] concerned flipped classroom studies in which students study online learning material prior to class, and apply this learning material on- and offline in class. The study by Kurt [33] was created on Edmodo, a social learning platform for teachers, students and school. Students were assigned to watch weekly lectures for the course via video podcasts using present.me, followed by an online quiz. In class, learning occurred through both face-to-face contact and practice-based interactive online tasks, mostly in pairs or groups. In Peterson's study [36], students were assigned to watch online lectures prior to arriving in class; students worked in class with a textbook on problem sets, followed by taking an online quiz at the end. In line with these studies [33,36], Safar and Alkhezzi's study [37] combinated face-to-face instruction and training with online instruction and training.

#### 4.3.1 Measured and reported positive effects on student engagement and learning outcomes

With respect to the treatment group (a flipped classroom) in Kurt's study [33], students' final examination scores (academic outcome) improved significantly compared to the scores for the students in the control group (traditional face-to-face learning). However, study [33] showed only a small impact of being in the flipped classroom on students’ cognitive engagement (sense of self-efficacy), among a sample of 62 s-year student teachers, compared to the students who engaged in face-to-face learning. On top of the measured effects, in Kurt's study [33], the students reported that the blended learning environment introduced (a flipped classroom) was more student-centered. They also evaluated the class as more positive and less stressful (personal outcome) in comparison with traditional face-to-face learning. In particular, the peer learning and the possibility of gaining knowledge and training online (before class, at their own pace and multiple times, if needed) was less stressful for them.

In Peterson's study [36], 43 students taking a statistics course valued blended learning in their flipped classroom as clearer and of higher quality, but also as more demanding, and not well-organized (cognitive engagement). Furthermore, being in the flipped classroom led to a lower grade-point average. However, students' cumulative exam score was moderately improved by being in the flipped classroom (academic outcome). The students in the control group engaged in traditional learning. It must be noted that after this study, the particular statistics course underwent a significant redesign, and in all eight times it was offered after that, students evaluated the flipped classroom significantly higher compared with the students engaging in traditional, face-to-face learning. The authors did not report whether this course redesign had an impact on learning outcomes as experienced by them.

Among a sample of 128 female undergraduate students who enrolled in a course on “Computing in Education” [37], blended learning (a combination of internet-based, web-based, computer-based, and face-to-face learning) showed a high positive influence on both the quality of their projects (academic engagement) and their final grade (academic outcome). In this study, blended learning was compared with face-to-face learning (the control group). In Safar and Alkhezzi's study [37] the number of online training courses attended, the tests taken, and the number of days absent (behavioral engagement) were positively and highly influenced by blended learning.

#### 4.3.2 Reported hindering factors

In Peterson's study [36], the author described the following challenges he had in implementing a flipped classroom. Creating video podcasts of all lectures meant a significant time investment for the author (teacher) involved. He had experienced a lack of institutional support, in that he was left to his own devices and had to find his own solution for supporting students. At the beginning, students expressed skepticism with regard to the flipped classroom concept; he had to convince students to be more responsible for their own learning, as this is essential for learning in a flipped classroom design. Later on, as students started to value this new learning concept, their skepticism faded away.

### 4.4 Online peer assessment

In the study of Svenningsen and Pear [38], unit tests were graded by either a teaching assistant or by two online peer reviewers by means of a Computer Aided Personalized System of Instruction (CAPSI). In CAPSI, students had to pass 20 unit tests and peer-assess 10 unit tests. The students in the treatment group engaged in face-to-face learning, but took unit tests and performed peer assessment online.

#### 4.4.1 Measured and reported positive effects on student engagement and learning outcomes

In their study, Svenningsen and Pear [38] found that although CAPSI had no impact on students' final examination score and a small impact on students' scores on critical thinking (academic outcome) among 364 students who attended the course on “Introduction to the University”, it had no impact on students’ applied critical thinking (cognitive engagement).

#### 4.4.2 Reported hindering factor

Although CAPSI did not have an effect on students' critical thinking, the experimental group's mean score for critical thinking was higher (9.33) than that of the control group (8.62). The authors suggested that the length of the experiment (13 weeks) was too short to show a positive effect on academic outcomes.

### 4.5 Online fun factor

In studies by Hew et al. [30a,30b], which had identical study designs, students in both the treatment group and the control group participated in online application tasks that addressed students’ need for autonomy, competence, and relatedness, in line with self-determination theory [45], while those in the treatment group also experienced game mechanics that were similarly supportive of the needs for competence, autonomy, and relatedness. A study by Wang [40] concerned a blended learning strategy based on augmented reality (AR). Students in the treatment group were provided with AR-based content for one of the learning topics, while those in the control group were provided with step-by-step video presentations on YouTube.

#### 4.5.1 Measured and reported positive effects on student engagement and learning outcomes

Hew et al.’s studies [30a,30b] compared gamified (*n* = 22) versus non-gamified (*n* = 44) blended learning for learning motivation, in a sample of 66 students participating in a course for designing questionnaires. Although both studies [30a,30b] found a positive and high impact of gamification on the number of forum messages students posted (behavioral engagement), only one study [30b] found a positive moderate impact on students' recall of factual knowledge. On the contrary, in the other study [30a], gamified blended learning had a negative impact on students' recall of factual knowledge (academic outcome). On top of the measured effects, in both studies [30a,30b], game mechanics were reported to be a powerful incentive that motivated students to work harder; comparison of students' activities with each other stimulated students to “keep up with the rest”, or to work harder than the rest (academic engagement).

In Wang's study [40], the AR-based blended learning strategy generated mixed results among 103 educational students. AR had a moderate negative impact on repeated measures of learner feedback (cognitive engagement), and a small negative impact on weekly grades (academic outcome). In this study, the AR-based blended learning strategy was concluded to be a fun factor for students. After removal of the AR-based supports, the students in the treatment group (*n* = 59) showed greater learning involvement in the activity and worked hard to finish their work. In contrast, only the advanced students and those with great interest in the course (video-editing), showed good learning motivation without the online learning supports on 10.13039/100024999YouTube.

#### 4.5.2 Reported hindering factors

Hew et al.’s two studies [30a,30b], which involved gamification, differed in their duration: 3 days (experiment 1) versus 18 days (experiment 2). According to the authors, the duration of the course in experiment 1 (3 days) might not have been long enough to have an effect on students' recall of factual knowledge.

In Wang's study [40], the authors mentioned the following hindering factors: 1) the shortage of experience with AR, 2) the slow internet speed at the learning environment, 3) the limited features of learner's mobile devices, 4) the small screen size of students' learning interfaces, and 5) the cognitive overload of AR-learning information.

### 4.6 Individualized intervention approach

Wong et al.’s study [41] made use of a monthly learning report by which students could compare themselves in terms of active learning behavior, online and in-class, with other students.

#### 4.6.1 Measured and reported positive effects on student engagement and learning outcomes

In Wong et al.’s study [41], sophomore students attended a technical course (*N* = 49) with a blended learning design. All students received a monthly learning report by e-mail, based on their learning behavior in the classroom and online. In line with the results, students received customized feedback and interventions from their tutors. Those in the experimental group (*n* = 27) saw individualized information in their monthly report, while students in the control group (*n* = 22) only saw the total group score for the class. Results showed that the individualized intervention approach had a high positive influence on motivation (cognitive engagement) and a moderate impact on academic achievement (academic outcome). On top of the measured effects, the authors reported that student-centered learning, real-time feedback, real-time intervention, customized feedback and intervention based on students' needs led to positive effects on students' self-efficacy (cognitive engagement).

### 4.7 To what extent does student engagement relate to learning outcomes, in blended learning?

In none of the 15 included studies, the authors hypothesized and researched whether dimensions of student engagement in blended learning were related positively to students’ learning outcomes.

## 5 Discussion

The aim of this systematic review was twofold 1) to research the effectiveness of blended learning designs with respect to student engagement and learning outcomes in higher education, as well as hindering factors, and 2) to examine to what extent student engagement relates to learning outcomes in blended learning.

The results generally show that over all included studies (*k* = 15, *N* = 1,428) blended learning had a moderate to high impact on the different dimensions of student engagement as addressed in this review: academic, behavioral, cognitive, and affective engagement [20,21], as well as on learning outcomes, regardless of its design features. In two studies, students’ academic learning outcomes were negatively influenced [30b,35], and in three studies [36,38,40], effects on academic outcomes generated mixed results (both positive and negative). It must be stipulated that in the latter three studies [36,38,40], the duration of the experiments was considered too brief to show an effect on learning.

In the current review, the following five blended learning designs were found to be beneficial for both student engagement and learning outcomes.

1. An online learning community of students and tutors meant to benefit peer learning and student-tutor interaction [28,29,31,32,34,35,39,42];
2. A flipped classroom that let students prepare their lessons online, (e.g., by videos or podcasts), then let students participate in class in off- and online application tasks, mostly in groups of students, to promote self-regulated learning [33,36,37];
3. Online peer assessment as a necessary part of the learning process [38];
4. An online fun factor meant as an incentive for students to learn more and work harder (e.g., by handing out badges or medals online, or by using Augmented Reality [30a,30b,^40^]);
5. An individualized intervention approach providing students with a monthly report of their on- and offline learning behavior and a customized intervention [41].

In the majority of studies, a blended learning design was compared with face-to-face learning. However, in three out of 15 included studies [30a,30b,^41^], two different blended learning designs were compared. In one study consisting of two experiments [30a,30b], an online fun factor was shown to increase students' behavioral engagement in a blended learning environment. It must be noted that with respect to academic outcomes, these studies [30a,30b] showed mixed results. In the third study, an individualized intervention approach in a blended learning environment was shown to have a moderate to high impact on both students’ cognitive engagement and their academic outcomes, compared to students experiencing an undifferentiated intervention approach in the blended learning environment [41].

None of the included studies measured whether and how student engagement in learning and learning outcomes were related. However, given the results in the current review, it is plausible that the student engagement measures studied would have been positively related to students’ learning outcomes, if this had been under research. Namely, all effects regarding student engagement were positive. Similarly, effects for learning outcomes were generally positive.

### 5.1 Strengths and limitations of this systematic review

A strength of this review was the inclusion or computation of effect sizes for the outcome measures in each study included in this review. As a result, Cohen's *d* was computed for student engagement and for learning outcomes, resulting in comparable statistics for student engagement and learning outcomes between studies. The effect sizes led to extra information concerning the impact of blended learning, on top of the significance levels that had been published earlier in the included studies (see also [24,25]).

The current review focused on (quasi-)experimental blended learning studies with a control group, published after 2009. The majority of the studies (11 out of 15 studies included) were conducted in Asia. One of these studies consisted of two experiments. Another three included studies concerned European (1) and Eurasian (2) contexts. Only one included study concerned an American context. Although the outcome measures in general were consistent across studies, we have to be aware that contextual variables concerning the student population on which studies in the current review mainly focused (Asian students) might have influenced the outcome measures used in this body of research.

Due to the heterogeneity in the outcome measures, we chose to systematically review the evidence instead of meta-analyzing it. A limitation of systematically reviewing the evidence is that, in contrast with a meta-analysis, it was not possible to measure moderator effects such as design features of blended learning, student population, or length of the experiment.

### 5.2 Recommendations for further research

The meta-construct of student engagement has shown to be a worthwhile means for getting insight into what makes blended learning “work” in higher education. However, the included studies all focused on only one or two dimensions of student engagement. Therefore, we highly recommend to conduct future research on the impact of blended learning on the different dimensions of student engagement (academic, behavioral, cognitive, and affective engagement) over time, and the impact of different blended learning approaches on all four dimensions of student engagement.

It is striking that affective engagement was measured in only one study [28]. Likewise, personal and social learning was measured in only one study [31], and none researched learning outcomes with regard to citizenship. However, additional effects on personal and social learning as reported (not measured) by the authors [33,39] indicate that blended learning has more potential effects than might be directly measurable. It is therefore recommended that blended learning studies focus on more dimensions of both student engagement and learning outcomes.

As already mentioned in the strengths and limitations section, the current systematic review mainly included Asian studies (11 out of 15). It would be worthwhile to know to what extent contextual differences influence the effect of blended learning in different student populations. It is therefore recommended that more (quasi-)experimental studies with a control group be conducted on other continents. Lastly, it is notable that, in contrast with the literature that claims students in STEM disciplines to benefit more from blended learning than non-STEM students, the data in this review do not confirm this. Actually, blended learning might be beneficial for all students with respect to their engagement and their learning outcomes.

## 6 Conclusion

In conclusion, the data in this review show that blended learning is a promising tool for facilitating both students’ engagement and their learning outcomes. Based on the current study, the following blended learning designs are recommended as a place to start when shifting from face-to-face learning to blended learning: 1) an online learning community of students and tutors for promoting peer learning and student-tutor interaction, 2) a flipped classroom in which students are asked to prepare their lessons online, prior to class (e.g., by videos or podcasts), followed by off- and online application tasks, mostly in groups of students, for promoting self-regulated learning and 3) online peer assessment. Furthermore, for lecturers who already teach their students in a blended learning format, both an online fun factor (e.g., by handing out badges or medals online, or by using Augmented Reality) and an individualized intervention approach (providing students with a monthly report of their on- and offline learning behavior and in line with their report, provide customized intervention) might serve as an incentive for their students to learn more successfully. In the current review, the different included studies are described, pulling out the elements of blended learning designs that have been shown to engage students (academically, behaviorally, cognitively, and affectively), and to benefit their learning outcomes, (academically, personally, socially, and with regard to citizenship). With this knowledge, lecturers and policymakers can introduce blended learning in higher education in an evidence-based way, so that they can design and monitor blended learning for both student engagement and learning outcomes. For future studies it would be noteworthy to research: 1) how students can be engaged, not only academically, behaviorally, and cognitively, but also affectively, and 2) how blended learning designs can impact student engagement along with their learning outcomes, not only academically, but also personally, socially, and with respect to citizenship.

## CRediT authorship contribution statement

**M. De Bruijn-Smolders:** Writing – review & editing, Writing – original draft, Visualization, Validation, Supervision, Software, Resources, Project administration, Methodology, Investigation, Funding acquisition, Formal analysis, Data curation, Conceptualization. **F.R. Prinsen:** Writing – review & editing, Writing – original draft.

## Data availability statement

All data required for this systematic review is yet mentioned in this manuscript.

## Funding

Research funded by the Research Centre 10.13039/100023749Urban Talent, 10.13039/100021096Rotterdam University of Applied Sciences, Rotterdam, the Netherlands.

## Declaration of competing interest

The authors declare the following financial interests/personal relationships which may be considered as potential competing interests: Monique de Bruijn-Smolders reports financial support was provided by Research Centre 10.13039/100023749Urban Talent, 10.13039/100021096Rotterdam University of Applied Sciences. If there are other authors, they declare that they have no known competing financial interests or personal relationships that could have appeared to influence the work reported in this paper.
